# Spare Parts Demand Forecasting Method Based on Intermittent Feature Adaptation

**DOI:** 10.3390/e25050764

**Published:** 2023-05-07

**Authors:** Lilin Fan, Xia Liu, Wentao Mao, Kai Yang, Zhaoyu Song

**Affiliations:** College of Computer and Information Engineering, Henan Normal University, Xinxiang 453007, China; fll@htu.edu.cn (L.F.); jxgzxglx@163.com (X.L.); 18336200890@163.com (K.Y.); songzhaoyouxiang@163.com (Z.S.)

**Keywords:** intermittent time series, deep learning, demand forecasting, transfer learning, spare parts management

## Abstract

The demand for complex equipment aftermarket parts is mostly sporadic, showing typical intermittent characteristics as a whole, resulting in the evolution law of a single demand series having insufficient information, which restricts the prediction effect of existing methods. To solve this problem, this paper proposes a prediction method of intermittent feature adaptation from the perspective of transfer learning. Firstly, to extract the intermittent features of the demand series, an intermittent time series domain partitioning algorithm is proposed by mining the demand occurrence time and demand interval information in the series, then constructing the metrics, and using a hierarchical clustering algorithm to divide all the series into different sub-source domains. Secondly, the intermittent and temporal characteristics of the sequence are combined to construct a weight vector, and the learning of common information between domains is accomplished by weighting the distance of the output features of each cycle between domains. Finally, experiments are conducted on the actual after-sales datasets of two complex equipment manufacturing enterprises. Compared with various prediction methods, the method in this paper can effectively predict future demand trends, and the prediction’s stability and accuracy are significantly improved.

## 1. Introduction

**Figure 1:**
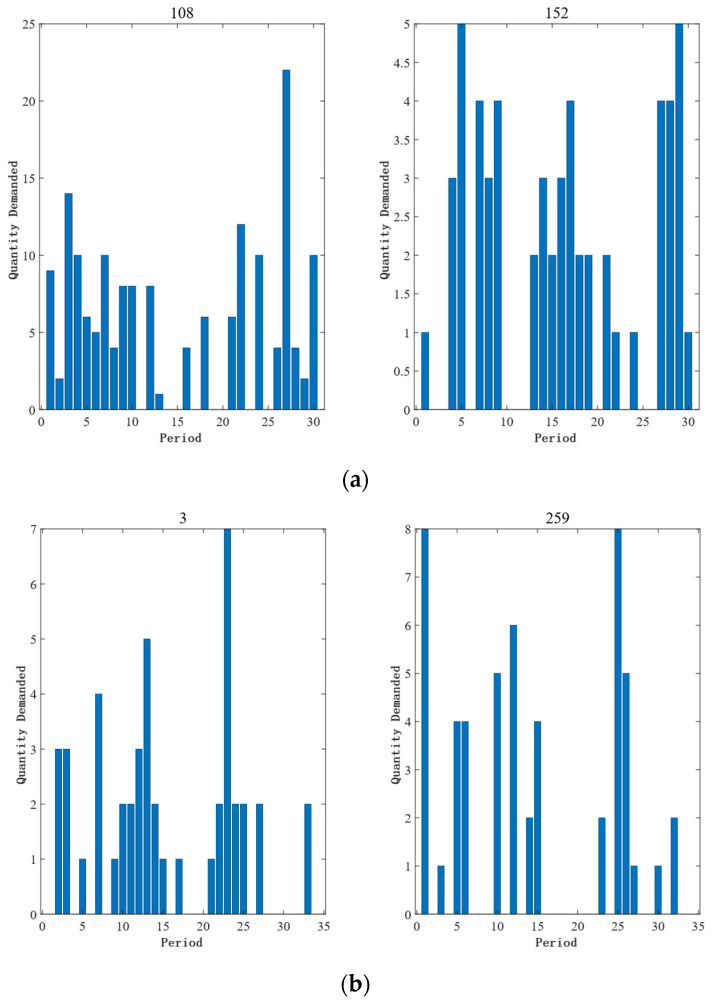
Intermittent time series data distribution: (**a**) 30-month number of sales for spare parts No. 108 and No. 152 in dataset 1; (**b**) 34-month failure counts for spare parts No. 3 and No. 259 in dataset 2.

Intelligent operation and maintenance can automatically learn the rules from massive after-sales data through machine learning, and efficiently provide a decision basis for the production and sales activities of an enterprise. Demand forecasting, as one of the key links, can effectively provide data support for the inventory arrangement of an enterprise. With the gradual emphasis on aftersales service quality, accurate demand forecasting has become an urgent problem for complex equipment-manufacturing enterprises. In actual application, the demand for spare parts is closely related to the new project launch, the intensity, and the environment of equipment use, which makes the aforementioned factors show obvious intermittency and fluctuation, as shown in Figure 1, bringing a great challenge to the accurate prediction of demand. However, from the cost point of view, complex equipment parts cost a lot of money in production and storage, which brings a huge burden to enterprises. Therefore, it is not only of academic value but also of practical significance to study how to achieve effective demand forecasting, and thus reduce the operating cost of enterprises.

At present, the forecasting methods of spare parts demand mainly focus on traditional time series forecasting methods, which include exponential smoothing [1], moving average [2], SVR [3], random forest [4], BP [5], etc. These methods are adapted to demand series with strong trends and periodicity. In fact, due to the influence of holidays, climate, and other factors, the demand for spare parts will show a certain trend and periodicity in time. However, the influence of the project’s sporadic plan, equipment usage intensity, and environment makes demand show obvious intermittency. This intermittency dilutes the trend and periodicity of the sequence itself to a certain extent, making traditional time series prediction methods ineffective. Therefore, applying the intermittent characteristics of the demand series to the forecasting model can effectively improve the forecasting performance. As one of the representatives, Croston et al. [1] divided the time series into demand size and demand time interval subseries and used the exponential smoothing method to forecast them, respectively, followed by the ratio of the two, as the demand forecasting value. Syntetos et al. [6] considered that Croston’s method directly used the ratio as the forecast value has a bias, for which a correction factor is proposed to smooth the ratio. Teunter et al. [7] found that the above methods do not update the demand forecast value when there is no demand for a long period, so they proposed to calculate the probability of demand occurrence first, then multiply the demand estimate to obtain the demand forecast value. These methods achieve good forecasting results when facing intermittent time series. However, they are limited by the problem of insufficient evolutionary information of a single series to further improve the forecasting effect. For collaborative prediction of multiple demand series, Montero-Manso et al. [8] applied the XGBoost machine learning framework [9] to the M4 competition; it learns structured information among multiple commodity demand series by generating encoded features for different series. Spyros et al. [10] pointed out that LightGBM [11], compared to XGBoost, can directly support sequence numbering and other features, reduces the workload of data processing and transformation, has significant advantages when oriented to multiple sequence prediction, and is widely used in M5 competitions. Muhaimin et al. [12] generated 72 different features based on data characteristics and used an RNN model to predict future demand, and the results showed that the method outperformed Croston et al.’s model. Thanks to the structured information between the demand series, these methods have more accurate prediction performance than previous methods. However, the sparse and widely varying distribution of complex equipment spare parts demand makes the prediction effect of these methods less stable.

The spare parts in the same equipment have the same intensity of use, which makes the various spare part demands have a certain degree of correlation in the equipment. However, complex equipment often has different models and different numbers of equipment working in different environments, which makes the correlation between the demands complicated, and ultimately makes it difficult for previous models to accurately extract common information between series. From the above analysis, it can be seen that the key to intermittent time series prediction is (1) how to represent the differences between the series and extract the association information between the demand series from the differences in order to further learn the common evolutionary law among spare parts demand; (2) how to integrate the temporal and intermittent characteristics of the demand series into the model in order to learn the inner evolutionary law of spare parts demand. Given this, this paper proposes a prediction method of intermittent feature adaptation based on transfer learning. Firstly, an intermittency metric is constructed based on the intermittency characteristics of the demand series, and a clustering algorithm is introduced to divide the originally disordered demand data into domains. Secondly, the intermittency and temporal characteristics of the demand series are combined to construct a weight vector, and the common evolution information among demands for spare parts is effectively learned by weighting the distance between the output features of each cycle between domains in order to improve the prediction effect. Finally, the experiments are conducted on the industrial big data complex equipment spare parts demand prediction competition dataset and the actual aftermarket data of a complex equipment manufacturing enterprise. The main contributions of the method in this paper can be summarized as follows.

(1) A domain partitioning scheme for intermittent time series is proposed, which is different from the existing classification methods for intermittent time series. The scheme adopts a clustering method to measure the differences in two dimensions: demand time and demand interval, thus enabling efficient and flexible differentiation of spare parts with different evolutionary trends.
(2) A domain adaptive algorithm for intermittent time series is proposed, which uses the intermittent and temporal characteristics of demand series to construct weight vectors, and learns the common information between domains by weighting the distance between domains, and it can improve the accuracy of demand prediction from both the special evolution law of demand series and the common evolution law between demand series, and no similar study has been found yet.

## 2. Related Theories

### 2.1. Hierarchical Clustering

Hierarchical clustering is a clustering algorithm using a tree structure, which is mainly divided into two clustering strategies, namely the top-down splitting strategy and bottom-up aggregation strategy; the second clustering strategy is used in this paper. This strategy starts by dividing all the elements involved in clustering into one class each, then merging the two most similar classes into a new class, and so on, until all elements are combined into a predetermined number of classes or other termination conditions are met, at which point the clustering algorithm ends.

### 2.2. Intermittent Time Series

The intermittency characteristic describes the sparsity, variability, and demand occurrence interval of the data, and represents the distribution characteristics of the data to some extent. Two currently accepted metrics are ADI and $CV^{2}$ [13], where ADI indicates the average demand interval of a sequence and $CV^{2}$ indicates the degree of variation of a sequence over time, and the calculation rules are as follows:

(1) $$ADI={\frac{T}{T_{occ}}}_{,}CV^{2}={\left(\frac{S}{\bar{x}}\right)}^{2}$$

where T and $T_{occ}$ denote the total number of periods of the sequence and the number of periods in which demand occurs, respectively; S is the standard deviation of nonzero demand; and $\bar{x}$ is the mean of nonzero demand. According to the above two indicators, the demand series can be classified into four different types, according to the following rules [13]:

(1) Smooth demand ($ADI<1.32,CV^{2}<0.49$);
(2) Intermittent demand ($ADI\geq 1.32,CV^{2}<0.49$);
(3) Irregular demand ($ADI<1.32,CV^{2}\geq 0.49$);
(4) Lumpy demand ($ADI\geq 1.32,CV^{2}\geq 0.49$).

### 2.3. Maximum Mean Discrepancy

The maximum mean discrepancy, *MMD* [14], is used to measure the distance between two distributions, and is often used as a loss function in domain adaptive models, which is defined as follows:

(2) $$MMD(X^{S},X^{T})=\left\|\frac{1}{\left|X^{S}\right|}\sum_{i=1}^{\left|X^{S}\right|}\phi \left(x_{i}^{S}\right)-\frac{1}{\left|X^{T}\right|}\sum_{j=1}^{\left|X^{T}\right|}\phi \left(x_{i}^{T}\right)\right\|$$

where $X^{S}$ and $X^{T}$ denote the source and target domains, respectively; and ϕ(X) is the kernel function. A Gaussian kernel function is used in this paper.

### 2.4. Predictive Performance Evaluation Metrics

In this paper, the mean absolute error (MAE), root-mean-square error (RMSE), and root-mean-square scalar error (RMSSE) [10,15] are used to evaluate the forecasting performance of the model, where MAE and RMSE are commonly used to evaluate the performance of time series forecasting and RMSSE is commonly used to evaluate the performance of intermittent time series forecasting, which is defined as follows:

(3) $$RMSSE=\sqrt{\frac{1}{h}\frac{{\sum_{i=t+1}^{t+h}\left(y_{i}-{\hat{y}}_{i}\right)}^{2}}{\frac{1}{t-1}\sum_{i=2}^{t}{\left(y_{i}-y_{i-1}\right)}^{2}}}$$

where t is the number of training sample periods, h is the number of forecast periods, $y_{i}$ is the true value at the time, and ${\hat{y}}_{i}$ is the estimated value at the time.

## 3. Intermittent Feature Adaptation-Based Spare Parts Demand Forecasting Method

**Figure 2:**
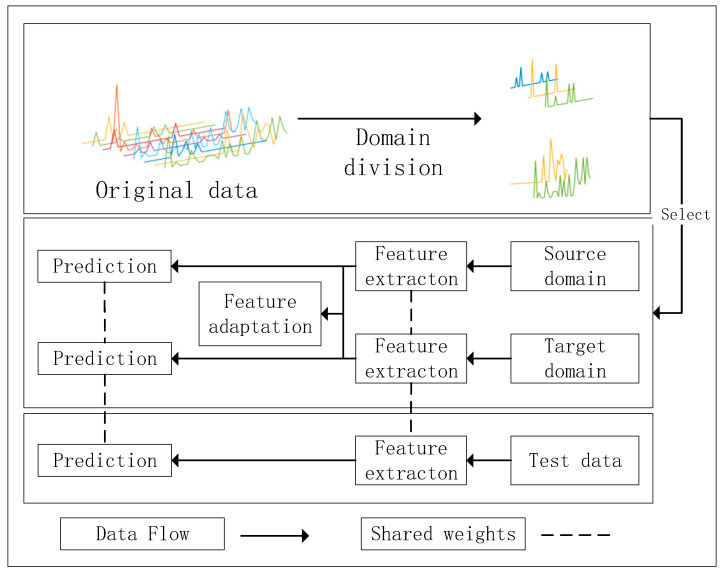
Flow chart of the method.

In this section, a novel intermittent time series prediction method based on depth feature transfer is proposed, which mainly contains the domain partitioning algorithm and domain adaptive algorithm for intermittent time series. The flow chart of the method in this paper is shown in Figure 2.

### 3.1. Domain Partitioning Algorithm for Intermittent Time Series

The essence of domain classification of intermittent time series is to use classification or clustering algorithms to divide them into different categories according to certain rules so that there are large differences between different categories of these series. The current classification algorithms mainly rely on the characteristics of the data to classify intermittent time series, such as Petropoulos et al. [16], who classified spare parts based on the statistical characteristics of the data of spare parts demand and chose a suitable prediction method for each category. However, the above methods have two shortcomings: (1) the classification is highly dependent on the seasonal, trend, statistical, and other characteristics of the series, and the final classification results are highly dependent on the chosen prediction method, i.e., good prediction results are available before finding a suitable classification boundary; (2) the classification is a supervised method, and it is difficult to intelligently split the unlabeled dataset, which in a practical environment, where the demand data are complex and variable, will bring a huge workload. In contrast, the clustering algorithm [17] can simply divide the data into several categories when a reasonable similarity index is available.

To realize the clustering of intermittent time series, the similarity index between series is constructed here first. The similarity between intermittent time series is different from general time series; the former is more concerned with the similarity of demand time within the same period between the series, while the latter is concerned with the similarity of the change in the quantity of spare parts demand in a certain period. In the relevant theoretical chapters, ADI and $CV^{2}$ are introduced, which reflect the average demand interval and fluctuation range of the series, and distinguish the intermittency degree of the series to some extent. However, they do not measure the relationship between the series from the perspective of demand occurrence time, so it is difficult to apply them to measure the similarity of intermittent time series. In summary, the key to constructing a good similarity index lies in the ability to accurately measure the relationship between demand times. To this end, this section introduces the concepts of zero-demand interval and last demand time, where zero-demand interval refers to how many cycles have passed since the last demand occurred, and last demand time refers to the cycle in which the last demand occurred. The following is the process of constructing the similarity index:

(1) Extend the original sequence $X_{i}={\left\{x_{ij}\right\}}_{j=1}^{T}$ as $X_{i}^{\prime }={\left\{x_{ij}^{\prime }\right\}}_{j=1}^{T}$, where $X_{i}={\left\{x_{ij}\right\}}_{j=1}^{T}$ denotes the ith sequence; T denotes the number of time series cycles; $X_{i}^{\prime }={\left\{x_{ij}^{\prime }\right\}}_{j=1}^{T}$ denotes the ith sequence after extension; $x_{j}^{\prime }=\left(x_{j},zp_{j},ld_{j}\right)$ denotes the extended element of the jth cycle, where $zp_{j}={\{}_{0\;\mathrm{if}\;x_{i}=0}^{j-last\;\mathrm{if}\;x_{j}>0,x_{last}>0,j<last}$ denotes the 0 demand interval and $ld_{j}$ denotes the last demand time.
(2) Calculate the similarity between the spare parts using the distance function $d(X_{i}^{\prime },X_{j}^{\prime })=MMD\left(X_{i}^{\prime },X_{j}^{\prime }\right)$.

In this section, the hierarchical clustering method is used to cluster the set of time series ${\left\{X_{i}^{\prime }\right\}}_{i=1}^{n}$, and the steps are as follows:

(1) Initially, consider each sequence ${\left\{X_{i}^{\prime }\right\}}_{i=1}^{n}$ in the set of demand series $X_{i}^{\prime }$ a class cluster.
(2) Calculate the distance between two clusters of classes, merge the two classes with the smallest distance into a new class, and delete these two classes. The distance calculation formula is as follows:

(4) $$D_{AB}=1/\left(\left|A\right|\times \left|B\right|\right)\sum_{X_{i}\in A}\sum_{X_{j}\in B}d\left(X_{i}^{\prime },X_{j}^{\prime }\right)$$

where A and B denote the classes involved in the distance calculation, and |A| and |B| denote the number of demand series in the class cluster.

(3) Repeat step 2 until the number of class clusters reaches a predetermined value.

Finally, all time series ${\left\{X_{i}^{\prime }\right\}}_{i=1}^{n}$ are clustered in k classes ${\left\{class_{i}\right\}}_{i=1}^{k}$, and as k domains ${\left\{domain_{i}\right\}}_{i=1}^{k}$.

### 3.2. Intermittent Feature Adaptation Algorithm

**Figure 3:**
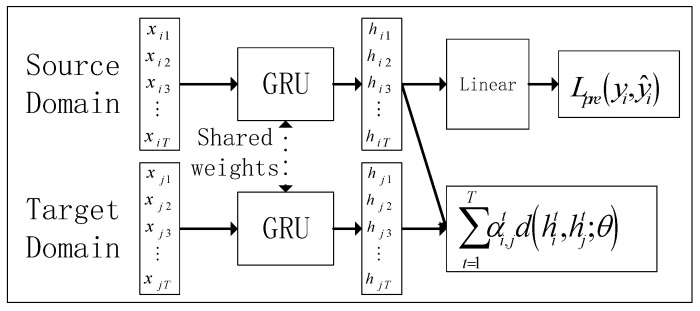
The framework of domain adaptive.

Most of the existing transfer algorithms adopt the strategy of parameter transfer, which requires a large dataset as the source domain; it is trained in the large dataset and then fine-tuned in similar small data, while the size of the spare parts demand dataset is usually small, and the distribution of different spare parts in terms of data characteristics varies greatly, making it difficult to apply the parameter transfer strategy to the problem in this paper. For this reason, this section adopts the strategy of domain adaptation [18] to realize the information transfer between domains, as shown in Figure 3. The core of domain adaptation lies in its loss function:

(5) $$Loss=\sum_{i=1}^{k}L_{pre}\left(y_{i},{\hat{y}}_{i}\right)+\sum_{i,j}^{i\neq j}L_{d}\left(h_{i},h_{j}\right)$$

The first half of the formula is the prediction loss, which guides the model to learn the future trend information of the sequence. The second half is the distance loss between domains, which reduces the discrepancy of depth features between domains and completes the information transfer from the source domain to the target domain.

However, the above formula only calculates the interdomain distance on the whole, without paying attention to the time-dependent relationship of the hidden layer output in the time series. Specifically, with GRU as the feature extractor, there is a temporal relationship between the output features corresponding to each cycle of the time series. How to learn this time-series relationship is the key to improve the prediction performance, so this paper introduces the temporal distribution matching algorithm TDM [19], which contains two main parts: the loss algorithm for constructing the distribution distance using the weight matrix and the algorithm for updating the weight matrix. Considering the intermittency of demand series, to make TDM applicable to intermittent time series, this section improves on TDM by integrating intermittent features with the weight matrix, so that it can learn the structured information between domains from the intermittency perspective.

TDM learns the temporal relationships between domains by using a weight matrix $\alpha \in R^{T}$ to represent the importance of the feature distances for each cycle, where T denotes the number of sequence cycles. By adjusting the weight matrix α, the domain feature differences can be dynamically reduced. When given a specific source domain $D_{i}$ and target domain $D_{j}$, the distribution loss is as follows:

(6) $$L_{tdm}(D_{i},D_{j};\alpha )=\sum_{t=1}^{T}\alpha_{i,j}^{t}d\left(h_{i}^{t},h_{j}^{t};\theta \right)$$

where $\alpha_{i,j}^{t}$ denotes the feature importance weights of $D_{i}$ and $D_{j}$ at t moments, and $h_{i}^{t}=\delta (x_{i}^{t},h_{i}^{t-1})$ is the GRU network hidden layer output. Combining the predicted loss yields the following target loss function:

(7) $$L\left(\theta ,\alpha \right)=\sum_{i}^{\left|k\right|}L_{pre}\left(y_{i},{\hat{y}}_{i}\right)+\lambda \frac{2}{k(k-1)}\sum_{i,j}^{i\neq j}L_{tdm}(D_{i},D_{j};\alpha )$$

The hyperparameter λ is used to balance the prediction loss and interdomain distance loss of the model.

In summary, it can be seen that the TDM learns the interdomain time series relationship by α weighting the hidden layer feature distance. At this time, how to update the parameter α becomes the key to the feasibility of the above method. The original text directly adopts the way of boosting. However, it has been analyzed in the introduction that intermittent time series should not only consider the temporality of the features, but also the intermittency of the demand series. Therefore, this section incorporates intermittent features into the boosting method, so that α can learn both the temporality and intermittency of features in the updating process and better realize the sharing of knowledge among domains.

After the domain partitioning of the original data using the algorithm in Section 2.1, the data within the domains still differ, and further mining the differences will help to learn the structured information in them in depth at this time. Specifically, when two specific domains are given, demand series in which the intermittent features are more similar and demand series with larger differences should have different degrees of importance in the process of reducing the differences in domain distributions. Given this, this section proposes the coefficient of intermittent differences β between the demand series auxiliary update parameter α, and it is calculated from the zero-demand interval sequence as well as the demand occurrence time series. The equation is as follows:

(8) $$\beta =sig\mathrm{mod}\left(\left|zp_{i}-zp_{j}\right|+\left|lp_{i}-lp_{j}\right|\right)$$

The rules of computing zp and lp are described in Section 2.1. β denotes the intermittent difference between domains, and it is combined with α to realize the learning of common knowledge between domains from both intermittent and temporal perspectives, i.e., when the intermittent difference between domains is larger, the temporal difference is larger. The focus is on reducing the difference among them and strengthening the learning of domain knowledge among them. β and α are combined in the following process:

(1) At the beginning of training, the hidden layer features change a lot. To make the update process of α more smooth, the pretraining times are set here as epoch0. Before the training times reach epoch0, α will be adjusted by the network adaptively; the adjustment rule is α=f(h)×β, where f(h) is the output of the fully connected layer.
(2) When the model has completed epoch0 pretraining times, the parameter α is updated by the boosting method; where α is not updated when the distribution loss in the new round does not increase compared to the previous round, and vice versa, the α is updated with the following rules:

(9) $$\alpha_{i,j}^{t,(n+1)}=\left\{ \begin{array}{l} \alpha_{i,j}^{t,(n)}\times G(d_{i,j}^{t,(n)},d_{i,j}^{t,(n-1)})\times \beta \;d_{i,j}^{t,(n)}\geq d_{i,j}^{t,(n-1)}\\ \alpha_{i,j}^{t,(n)}\;\;\;\;\;\mathrm{other} \end{array}\right.$$

where $G\left(d_{i,j}^{t,(n)},d_{i,j}^{t,(n-1)}\right)=(1+\sigma (d_{i,j}^{t,(n)}-d_{i,j}^{t,(n-1)}))$, σ is the sigmod function.

## 4. Experimental Analysis

To verify the validity of the proposed method, we conducted experiments on the spare parts demand data (Dataset 1) of Zoomlion enterprises provided by the “Fifth National Industrial Internet Data Innovation Application Competition” (https://www.industrial-bigdata.com/Competition, accessed on 1 April 2023). The practicality of the model was verified on the spare parts aftersales data of complex manufacturing enterprises (Dataset 2); it is unpublished data. The experimental hardware environment consisted of an i7-12700h processor, RTX3060 6 g graphics card, and 64 g ddr4 memory, and the software environment comprised Matlab 2018 and Python 3.8.

### 4.1. Dataset Introduction

Dataset 1 contains historical sales data of 1200 spare parts for a total of 30 months from January 2018 to June 2020, which includes data of spare parts in use quantity, equipment working hours, equipment type, and other attributes. Dataset 2 contains 34 months of spare parts sales data, which includes spare part numbers, spare part failures, and other attributes. Details are shown in Table 1.

Since this paper starts the research of spare parts demand prediction based on the characteristics of sequence interval, the demand series with larger demand intervals were filtered out according to the rule of ADI≥1.32, in which a total of 524 demand series were filtered out from Dataset 1 and a total of 858 spare parts are filtered out from Dataset 2.

### 4.2. Comparison Method

The methods compared in the experiments can be divided into three categories, which are traditional methods, machine learning methods, and deep learning methods, and the details of the compared methods are shown in Table 2.

In the experiments, the number of GRU layers was set to 3 and the hidden layer size was set to 128 for the model in this paper. The number of LSTM layers was 2 and the hidden layer size was set to 128. All model hyperparameters were searched by the grid to the optimum.

For Dataset 1, the data from month 1 to month 30 were used as the training set, month 2 to month 29 as the validation set, and month 3 to month 30 as the test set. For Dataset 2, the data from month 1 to month 32 are used as the training set, month 2 to month 33 as the validation set, and month 3 to month 34 as the test set.

### 4.3. Analysis of the Results of Intermittent Time Series Domain Segmentation Algorithm

**Figure 4:**
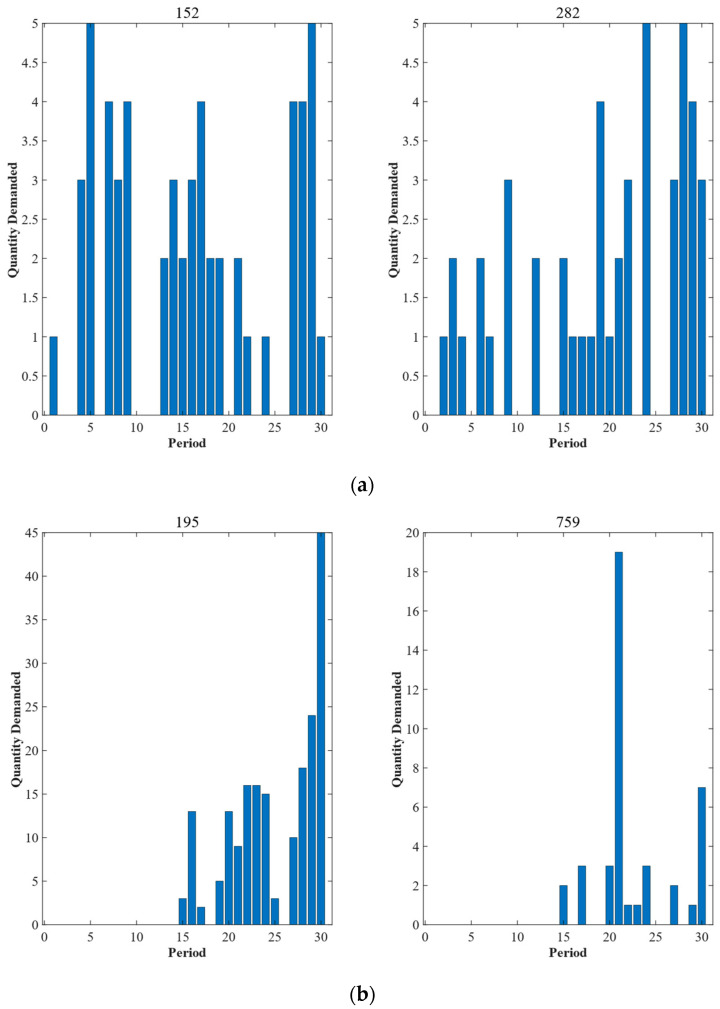
Domain division results. (**a**) Quantity demand of spare parts No. 152 and No. 282 in domain A. (**b**) Quantity demand of spare parts No. 195 and No. 759 in domain B.

Dataset 1 and Dataset 2 are divided into different domains, where the effect of Dataset 1 is shown in Figure 4. From Figure 4, it can be seen that the demand within the domain has greater similarity in intermittency and temporal order, while the demand between the domains has greater differences. For example, spare parts No. 195 and No. 759 in domain B both have an upward trend in month 15, and do not generate demand from month 1 to month 14. For spare parts No. 152 in domain A and No. 195 in domain B, there is a significant difference in the timing of the peak demand trend and in the time when demand did not occur.

### 4.4. Analysis of Intermittent Feature Adaptation Algorithm Results

**Figure 5:**
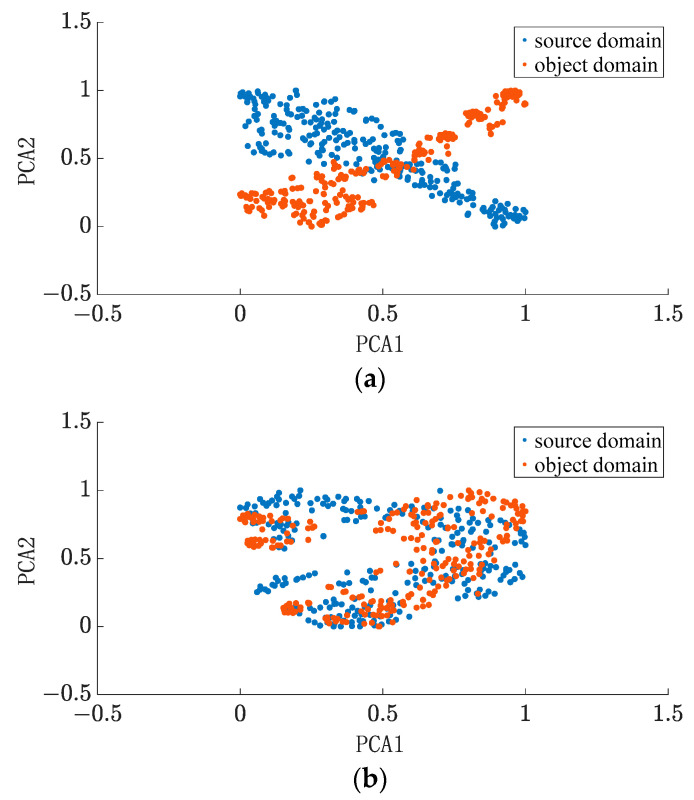
Comparison of feature distribution before and after adaptation: (**a**) before domain adaptation; (**b**) after domain adaptation.

The goal of the domain adaptation algorithm is to reduce the difference in depth features between domains, to achieve the extraction of common information, and finally, to improve the prediction effect. The feature distribution of Dataset 1 before and after adaptation is shown in Figure 5, and it can be clearly seen that feature adaptation can significantly reduce the distribution distance between domains.

**Figure 6:**
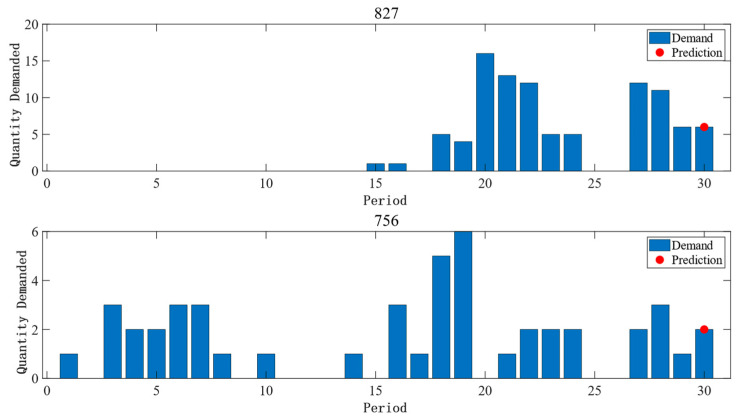
Trend and forecast results for demand for spare parts No. 827 and No. 756.

**Figure 7:**
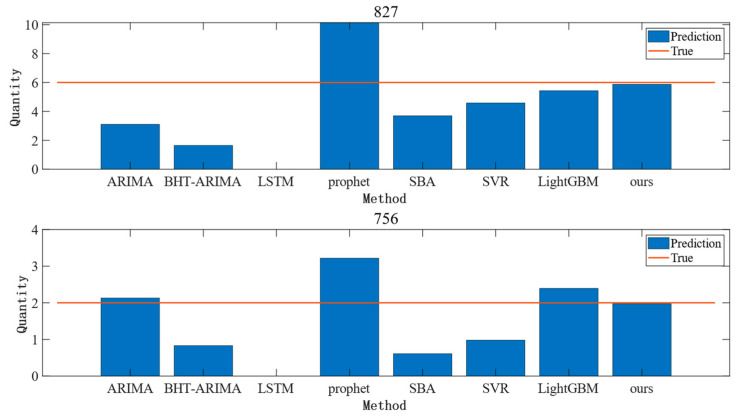
Comparison of the predicted results of spare parts No. 827 and No. 756.

After feature adaptation, the prediction effect of Dataset 1 is shown in Figure 6. Among them, spare part No. 827 and spare part No. 756 belong to different domains. It can be seen that spare part No. 827, as a whole, has a low number of demands, and no demand is generated in the beginning period, while spare part No. 756 has a more stable demand interval and has demand generated most of the time. To further address the contribution of common information to accurate forecasting, common forecasting methods are introduced here for the comparison of forecasting results, as detailed in Figure 7. In the experiment here, the demand information for the first 29 months of spare parts from Dataset 1 is used to forecast the demand for the 30th month. It can be seen in Figure 6 and Figure 7 that traditional methods such as ARIMA have good prediction results for a stable demand sequence such as spare part No. 756, indicating that traditional methods can still achieve good prediction results when the single sequence itself contains sufficient information on the law of demand evolution. In the face of spare part No. 827 and other spare parts with a small number of demand occurrences, the prediction effect of the previous method is not satisfactory, but the method of this paper can achieve a more stable prediction effect at this time. This indicates that the method of this paper makes full use of the information of the common evolution law among the demand series; it makes up for the shortcoming of insufficient information on the demand for a single spare part, and thus has a more stable prediction effect than other prediction methods.

**Figure 8:**
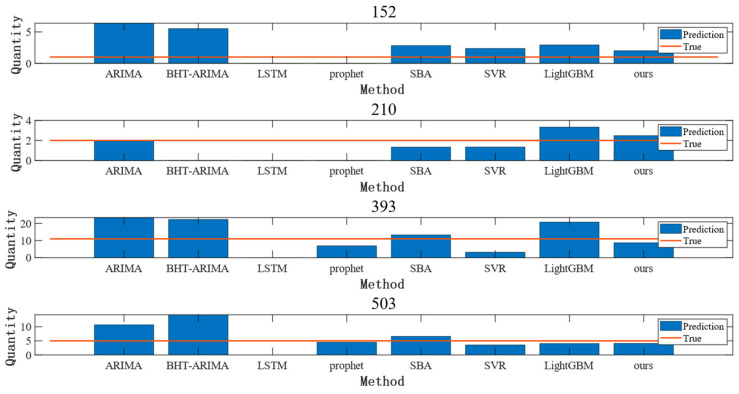
Comparison of the predicted results of spare parts No. 152, No. 210, No. 393, and No. 503.

Accurate prediction of intermittent time series is very difficult, and for most spare parts, the method in this paper has difficulty in achieving the prediction shown in Figure 7, while Figure 8 better represents the general level of prediction. From Figure 8, it is clear that spare parts No. 210, No. 393, and No. 503 are situations where the traditional methods outperform the methods in this paper. However, there are large deviations in the prediction results of these methods for some parts. Compared with other methods, the method in this paper is more stable in prediction results. Moreover, in the actual operating environment, stable spare parts can effectively avoid extreme shortage and redundancy situations, thus reducing the economic loss of the enterprise.

### 4.5. Ablation Experiments

The method in this paper consists of an intermittent time series domain partitioning algorithm and a domain adaptive algorithm incorporating intermittent features. To further verify the necessity of each part of the proposed method in this paper, the following ablation experiments are therefore designed in this section:

Option 1: putting all the data directly into the base model GRU without dividing the domain, as well as without using the domain adaptive algorithm.

Option 2: only the domain adaptive algorithm is used, and no intermittent difference coefficient is used.

The results are shown in Table 3. From the table, we can see that compared with the baseline model GRU, only the domain adaptive algorithm achieves advantages in all indexes, which indicates that using the common information among spare parts demands can improve the demand forecasting effect. After incorporating the intermittent difference coefficient β in the domain adaptive algorithm, the forecasting effect is further improved, which proves that using the intermittent feature to improve the performance of the domain adaptive model is rationality.

In summary, the model in this paper can start from the common information among spare parts demands and the intermittent characteristics of the sequence to explore the essential characteristics of the demand series, and then have better prediction performance.

### 4.6. Comparison Experiment

In actual business, due to the long manufacturing cycle and long delivery time of complex equipment spare parts, the demand for a longer period in the future needs to be evaluated. Therefore, to verify the practicality of the method in this paper, this experiment uses the historical demand quantity to predict the demand for the next three months and compares seven commonly used prediction methods, and the experimental results are shown in Table 4.

From Table 4, it can be seen that the method in this paper is the best in all indicators, while the SBA method has a better result in Dataset 1 and a less obvious effect in Dataset 2. It further shows that the method in this paper has a more stable prediction effect when dealing with different datasets because it makes full use of the common information among the series.

## 5. Concluding Remarks

In this paper, based on intermittent time series, a prediction method with intermittent feature adaptation is proposed. The method effectively divides multiple fittings into different subsource domains while combining the temporal information of demand series and intermittent features to fully exploit the common information among domains, which improves the prediction effect of the model in the face of sparse demand. The experimental results show that the method in this paper can effectively improve the demand prediction accuracy and stability, as well as providing reliable guidance for enterprises’ production preparation.

In actual environments, some spare parts are online for short periods, which leads to a lack of abundant historical demand data, while the relevant staff are more interested in knowing the confidence intervals of future demand. Therefore, in the next step, we plan to study the demand prediction problem of new online spare parts and provide reliable support for the prediction of new online spare parts demand by introducing the demand information of similar spare parts, while at the same time combining transfer learning and probability distribution to give confidence intervals for demand prediction in order to provide a more detailed decision basis for spare parts managers.

## Figures and Tables

**Table 1:** Data attributes.

Dataset Name	Periods	Number of Spare Parts	Attributes
Dataset 1	30	1200	Quantity, equipment working hours, equipment type
Dataset 2	34	999	Part numbers, number of spare part failures

**Table 2:** Comparison method.

Method Type	Method Name
Traditional Method	SBA [6], ARIMA
Machine Learning	SVR [3], BHT_ARIMA [20], Prophet [21], LightGBM [11]
Deep Learning	LSTM [22]

**Table 3:** Comparison table of ablation experiments.

Option	Dataset 1			Dataset 2		
	MAE	RMSE	RMSSE	MAE	RMSE	RMSSE
Option 1	0.3477	0.6382	0.3082	0.1476	0.2853	0.0445
Option 2	0.3414	0.6324	0.2986	0.1179	0.2786	0.0458
Ours	0.3297	0.5996	0.2813	0.1058	0.2685	0.0399

**Table 4:** Comparison of performance indicators of different methods.

Method	Dataset 1			Dataset 2		
	MAE	RMSE	RMSSE	MAE	RMSE	RMSSE
SBA	0.3392	0.6365	0.2829	0.1606	0.2878	0.0432
ARIMA	0.3939	0.7081	0.3842	0.1391	0.3383	0.0484
SVR	0.3515	0.6530	0.3226	0.1184	0.2700	0.0414
prophet	0.5806	0.8320	0.4779	0.1627	0.2945	0.0479
BHT_ARIMA	0.7799	1.1119	0.6965	0.4006	0.8196	0.1039
LightGBM	0.3801	0.6094	0.3246	0.1482	0.2785	0.0421
LSTM	0.6718	0.8143	0.5648	0.1669	0.2746	0.0434
Ours	0.3298	0.5997	0.2814	0.1058	0.2686	0.0400

## Data Availability

Not applicable.
